# Prenatal Chlorpyrifos Exposure Induces Ovarian Toxicity in Rat Offspring: Modulatory Potential of Curcumin

**DOI:** 10.3390/toxics14090820

**Published:** 2026-09-15

**Authors:** Gonca Gülsoy, Dilek Sağır

**Affiliations:** 1 Department of Environmental Health, Institute of Postgraduate Education, Sinop University, Sinop 57000, Türkiye; gonca_5117@hotmail.com; 2 Occupational Health and Safety, Faculty of Health Sciences, Sinop University, Sinop 57000, Türkiye

**Keywords:** chlorpyrifos, organophosphate pesticide, prenatal exposure, ovary, stereology, oxidative stress, curcumin

## Abstract

**Background:** Chlorpyrifos (CPF) is a widely used organophosphate pesticide that can reach the developing organism during gestation. The finite ovarian follicle pool is established early, yet prenatal CPF effects on it remain poorly characterized by unbiased quantitative methods. This study evaluated the developmental ovarian toxicity of prenatal CPF exposure and the modulatory potential of curcumin in rat offspring. **Methods:** Pregnant Wistar rats were assigned to Control, Sham, CPF (5 mg/kg/day), CUR (100 mg/kg/day), and CPF + CUR groups (five dams per group). Anogenital distance was recorded at birth (PND1). Female offspring were examined at postnatal day 28 using ovarian weight, histopathology, unbiased stereological follicle quantification, cortical volume estimation, serum anti-Müllerian hormone (AMH), serum cholinesterase activity, and biochemical markers of ovarian oxidative status and inflammation. The litter was the unit of analysis (*n* = 5 litters per group). **Results:** Prenatal CPF exposure produced marked, quantifiable alterations in ovarian structure, including decreased cortical volume, structural disorganization, vascular alterations, and follicular degeneration. Primordial, preantral, and antral follicle counts were significantly reduced versus controls (*p* < 0.0001), accompanied by significantly reduced ovarian weight and a shorter anogenital distance at birth. Serum AMH was lower in CPF-exposed offspring, and serum cholinesterase activity was reduced. In the curcumin co-treatment group, changes were partially less pronounced, with better-preserved follicular architecture and higher cortical volume than the CPF group. Ovarian oxidative and inflammatory activity increased with CPF exposure and was less marked with curcumin co-treatment. Reproductive-hormone measurements, including AMH, were not analytically validated in-house and are reported as exploratory. **Conclusions:** Prenatal CPF exposure induced structural and biochemical alterations consistent with developmental ovarian toxicity in rat offspring. Curcumin co-administration was associated with partially less pronounced changes, suggesting a modulatory rather than a fully protective role. Given the limited number of litters, findings should be interpreted with caution.

## 1. Introduction

Environmental exposure to organophosphate pesticides such as chlorpyrifos (CPF) during pregnancy is an increasing public-health concern for the developing organism. Among the potentially vulnerable targets, the ovary is a highly dynamic organ whose structural organization is established during early developmental stages. The primordial follicle pool, which underlies female reproductive capacity, is established during the perinatal and early postnatal period in rodents and constitutes a finite ovarian reserve that progressively declines throughout reproductive life [1,2]. The proper organization of follicular architecture and cortical structure is therefore essential for maintaining ovarian integrity and long-term reproductive potential. Disruptions occurring during critical developmental windows may result in permanent alterations in ovarian morphology, follicular dynamics, and tissue organization [3,4].

CPF has been associated with developmental toxicity in multiple tissues [5,6]. Mechanistically, it induces oxidative stress, disrupts antioxidant defense systems, and triggers inflammatory responses, thereby affecting cellular homeostasis [5,7,8,9]. These mechanisms are considered key contributors to CPF-induced tissue damage, particularly during sensitive developmental periods.

Several experimental studies have demonstrated that CPF exposure may adversely affect reproductive tissues. In rats, CPF has been reported to alter hormonal balance, impair follicular development, and induce structural damage in ovarian tissue [4,6,10,11]. In addition, CPF-induced oxidative stress and inflammatory responses have been associated with ovarian injury and increased follicular atresia in rats, consistent with broader evidence that insecticides disrupt follicular development and ovarian function [7,12,13]. Despite these findings, the effects of prenatal CPF exposure on early ovarian structural organization, particularly at the level of follicular populations and cortical volume, remain incompletely understood.

Curcumin, a natural polyphenolic compound derived from Curcuma longa, has attracted considerable attention due to its antioxidant and anti-inflammatory properties [14,15]. Experimental studies have demonstrated that curcumin can mitigate oxidative stress, regulate inflammatory responses, and protect tissue integrity under various pathological conditions [16,17]. In the context of reproductive biology, curcumin has been reported to influence ovarian structure and follicular reserve and to reduce tissue damage in rat and mouse models and in vitro [17,18,19]. These observations suggest that curcumin may have modulatory potential in the ovary under adverse conditions, although the evidence remains limited.

Despite these observations, there is limited information regarding the impact of prenatal CPF exposure on early ovarian morphology and structural organization. In particular, stereological evaluation of follicular populations and cortical volume in this context has not been sufficiently explored. Therefore, the present study aimed to characterize the developmental ovarian toxicity of prenatal CPF exposure in rat offspring, using stereological and histomorphological assessment, and to evaluate the potential modulatory role of curcumin.

## 2. Materials and Methods

### 2.1. Animals and Housing

The experimental cohort comprised 25 female and 25 male Wistar albino rats, each weighing 210–250 g, supplied by the Ondokuz Mayıs University Experimental Animal Research and Application Center. Throughout the study, room temperature was maintained at 19–22 °C and relative humidity at 55–60% under a 12 h light/12 h dark schedule. Standard chow and water were available ad libitum.

### 2.2. Mating, Group Allocation, and Exposure Protocol

After mating, females were checked for a vaginal plug and for sperm in vaginal smears. The day on which either finding was detected was recorded as gestational day 0 (GD0). Because each female was paired and followed separately, GD0 and parturition did not occur on the same calendar day for all animals. All 25 rats with confirmed pregnancy entered the experiment. Using body weight as a stratification variable, dams were randomly distributed among the Control (C), Sham, Chlorpyrifos (CPF), Curcumin (CUR), and CPF + CUR groups, with five dams in each group. Predefined exclusion criteria were signs of illness or deteriorating general health, and miscarriage or preterm delivery after confirmed pregnancy; any dam meeting either criterion was to be excluded from the study. No dam met these criteria after allocation, and litter size was not standardized by culling.

CPF (5 mg/kg/day; Sigma-Aldrich, St. Louis, MO, USA; Cat. No. 45395) and curcumin (100 mg/kg/day; Sigma-Aldrich, St. Louis, MO, USA; Cat. No. 239802) were prepared in corn oil. From GD0 through parturition, the relevant treatment was delivered once daily by oral gavage at 1 mL/kg body weight. For combined exposure, curcumin was given first and CPF was given 5 h later. Dams in the Control group were left untreated, whereas those in the Sham group received corn oil alone (1 mL/kg) over the same interval. Selection of 5 mg/kg/day CPF was informed by rat studies in which gestational or perinatal administration of 0.3–5 mg/kg/day produced maternal, developmental, or cholinesterase-related effects [10,20,21]. Thus, this dose was used as a biologically active experimental exposure and should not be interpreted as a regulatory NOAEL or as representative of usual human environmental exposure.

### 2.3. Offspring Monitoring and Selection

At spontaneous delivery, the number and sex of pups in each litter were documented, and all pups were weighed using a digital balance. Anogenital distance (AGD) was measured on postnatal day 1 (PND1) using a calibrated digital caliper as the linear distance between the center of the anus and the base of the genital papilla. Caliper accuracy was verified against a 10 mm certified reference block before each measurement session; the caliper had a resolution of 0.1 mm. Landmark identification (anus center to genital papilla base) followed a fixed protocol applied identically across all pups and litters. To account for differences in body size, the anogenital distance index (AGDi) was calculated for female offspring as AGD (mm)/body weight (g)^(1/3). AGD measurements were performed without access to group allocation information. The offspring were subsequently maintained under the stated housing conditions and checked for survival through PND28. Three female pups were selected at random from every litter for ovarian assessment at PND28. Their measurements were averaged before analysis so that each litter contributed one value (Section 2.8).

Because exposure was assigned to pregnant dams rather than to individual pups, the litter defined the experimental unit. All offspring-level observations were first averaged within litters, and the resulting five litter means per group were entered into the statistical analyses.

### 2.4. Gonadosomatic Index

At PND28, both ovaries were carefully dissected, cleared of adhering tissue, and weighed individually on an analytical balance (Shimadzu ATX224; readability 0.1 mg, Shimadzu Corporation, Kyoto, Japan). Relative ovarian mass was expressed as the gonadosomatic index (GSI), which was derived from the combined ovarian weight and body weight as follows [22]:

GSI = (*GA*/*TA*) × 100, where GA denotes the combined weight of the right and left ovaries and TA denotes body weight.

### 2.5. Ovarian Histopathology

Ovaries allocated to histology were kept in 10% neutral buffered formalin for 48 h and then processed routinely for hematoxylin and eosin (H&E) staining. Under light microscopy, sections were evaluated for haemorrhage, oedema, congestion, leukocyte infiltration, and follicular degeneration. Lesion extent was graded semiquantitatively on a four-point scale: 0, absent; 1, involving <33% of the examined area; 2, involving 33–66%; and 3, involving >66%. Three representative microscopic fields were evaluated for each animal, and the mean of the three field scores was used to obtain the animal-level score for each histopathological parameter. Group identity was concealed during assessment by assigning non-descriptive sample codes, and all scoring was completed without access to the allocation information.

### 2.6. Design-Based Stereology

#### 2.6.1. Optical-Fractionator Estimate of Follicle Number

Total ovarian follicle number was quantified with the optical fractionator. A systematic random series representing one of every two sections was examined; this yielded approximately 40 sections per tissue block (nominal cut thickness 25 μm; see measured thickness below). Each of the three right ovaries per litter was sampled and counted separately, and the three estimates were averaged so that each litter contributed a single follicle-number value. Stereo Investigator 9.0 (MBF Bioscience (MicroBrightField), Williston, VT, USA) was used for sampling and counting. The counting frame covered 6400 μm^2^, the sampling step area was 25,000 μm^2^, and the resulting area sampling fraction (asf) was 0.256. A 10 μm optical dissector height was used, with a 3 μm upper and an ~2.4 μm lower guard zone. Although sections were cut at a nominal thickness of 25 μm, the mean measured (mounted) section thickness was ~15.4 μm owing to processing-related shrinkage, giving a thickness sampling fraction (tsf) of approximately 0.65 (10/15.4). Counting was performed with a 63× oil-immersion objective (numerical aperture 1.40).

Follicular stages were assigned from the number and organization of granulosa-cell layers surrounding the oocyte [23]. A follicle was eligible for counting only when the sampled oocyte nucleus contained a visible nucleolus. Counts were made through unbiased counting frames placed according to the systematic-random sampling scheme. Follicle number was estimated with the equation below:

*N* = ΣQ^−^ × (1/*ssf*) × (1/*asf*) × (1/*tsf*)

In this expression, ΣQ^−^ is the number of sampled oocyte nuclei; *ssf*, *asf*, and *tsf* correspond to the section, area, and thickness sampling fractions, respectively.

#### 2.6.2. Cavalieri Estimation of Ovarian Compartment Volumes

Volumes of the ovarian cortex and medulla were derived with the Cavalieri estimator. From the systematic-random series, every fourth section was selected for this analysis. Images were acquired at 4× with an Olympus BX43 microscope (Olympus Corporation, Center Valley, PA, USA), and point counting was carried out in ImageJ Version 1.54 (National Institutes of Health, Bethesda, MD, USA). Pilot measurements established an area per point, *a*(*p*), of 10,000 μm^2^. Compartment volume was then estimated using the following Cavalieri equation [24,25]:

*V* = Σ*P* × *a*(*p*) × *t* × *k*

Here, ΣP is the sum of points intersecting the compartment of interest, a(p) is the tissue area represented by one point, t is the mean measured section thickness (~15.4 μm), and k is the section-sampling interval.

Precision and inter-animal variability of the stereological estimates were summarized by the coefficient of error (CE) and coefficient of variation (CV), respectively.

### 2.7. Sample Collection and Biochemical Measurements

At PND28, deep anaesthesia was induced with intraperitoneal ketamine (90 mg/kg) and xylazine (10 mg/kg). Immediately before euthanasia, 3–4 mL of blood was collected by cardiac puncture, representing near-complete terminal exsanguination. Animals were then perfused intracardially with 0.9% saline; no perfusion fixative was used.

Serum was separated by centrifuging the blood at 2000 rpm for 15 min at 4 °C and was kept at −80 °C pending analysis. The right ovaries of the three selected offspring per litter were each immersion-fixed in 10% neutral buffered formalin and processed individually for the histological and stereological procedures described in Section 2.5 and Section 2.6. Serum follicle-stimulating hormone (FSH), estradiol (E2), prolactin, and progesterone were quantified with rat ELISA kits from Shanghai Sunred Biological Technology Co., Ltd. (Shanghai, China): 201-11-0183, 201-11-0175, 201-11-0191, and 201-11-0742, respectively. Serum samples were analyzed without pre-dilution, and no dilution factor was applied to the calculated concentrations. Manufacturer-reported performance characteristics included an FSH range of 0.25–60 mIU/mL, sensitivity of 0.202 mIU/mL, and intra- and inter-assay coefficients of variation below 9% and 11%, respectively; the reported progesterone sensitivity was 1.115 ng/mL. Serum anti-Müllerian hormone (AMH) was measured with a rat-specific ELISA (CUSABIO Technology LLC, Wuhan, China; Cat. No. CSB-E11162r) according to the manufacturer’s instructions; the kit’s working range is 0.2–15 ng/mL with a stated sensitivity of 0.1 ng/mL. Because AMH immunoassays are not harmonised across platforms, absolute values are assay-dependent and not directly comparable with reference ranges obtained on other assays. Full kit specifications, the intra-assay precision computed from duplicate wells, and the outstanding validation items are compiled in Supplementary Table S9. These immunoassays were not validated in-house; the reproductive-hormone and AMH data are therefore reported as exploratory (Supplementary Table S4).

Serum cholinesterase activity was measured colorimetrically with the Elabscience kit E-BC-K174-M (Elabscience Biotechnology, Wuhan, China), an Ellman-based assay using acetylthiocholine as substrate and DTNB as chromogen (read at 412 nm) [26]; the manufacturer’s protocol was followed. The kit does not incorporate selective cholinesterase inhibitors (e.g., BW284c51 or ethopropazine), and serum cholinesterase in rodents is predominantly butyrylcholinesterase; the assay therefore does not discriminate between acetylcholinesterase and butyrylcholinesterase, and the reported activity should be interpreted as total serum cholinesterase. Erythrocyte and brain acetylcholinesterase, the matrices conventionally used in organophosphate toxicology, were not assessed.

For each litter, the left ovaries of three female offspring were pooled (~45 mg tissue) and homogenized in cold PBS (0.01 M, pH 7.4, with a protease-inhibitor cocktail) at 1:9 (*w*/*v*; ~405 µL), on ice and without detergent. Homogenates were centrifuged at 12,000× *g* for 15 min at 4 °C, and the supernatant (~450 µL per litter) was aliquoted to avoid freeze–thaw cycles (tissue ELISAs 160 µL; MDA 100 µL; GSH/T-SH 80 µL; SOD/CAT/GPx 30 µL).

All oxidative-stress, thiol, and cytokine results were normalized to wet tissue weight and expressed per gram of tissue; no protein-based normalization was applied. Superoxide dismutase (SOD) activity was assayed as described by Sun et al. [27], catalase (CAT) according to Aebi [28], glutathione peroxidase (GPx) according to Lawrence and Burk [29], and malondialdehyde (MDA) according to Placer et al. [30]. Reduced glutathione (GSH) content, determined as the nonprotein sulfhydryl fraction, and total sulfhydryl (T-SH) levels were measured using Ellman’s reagent according to Sedlak and Lindsay [31]. Results were expressed as SOD and GPx in U/g tissue, CAT in katal/g tissue, MDA and GSH in nmol/g tissue, and T-SH in µmol/g tissue.

Ovarian NF-κB, IL-1β, IL-6, and TNF-α were quantified in duplicate by sandwich ELISA using kits from Bioassay Technology Laboratory (BT LAB, Shanghai, China): NF-κB (Cat. No. E0287Ra), IL-1β (Cat. No. E0119Ra), IL-6 (Cat. No. E0135Ra), and TNF-α (Cat. No. E0764Ra), each performed according to the manufacturer’s protocol.

### 2.8. Statistical Procedures

Data are presented as mean ± standard deviation (SD) for continuous variables and as median (interquartile range, IQR) for ordinal histopathological scores. The litter was considered the experimental unit, and the five litter-level observations in each group constituted the independent observations for all statistical analyses (*n* = 5 litters/group). Normality of continuous variables was assessed using the Shapiro–Wilk test, and homogeneity of variances was evaluated using Levene’s test. Variables satisfying the assumptions for parametric analysis were analyzed using one-way analysis of variance (ANOVA), followed by Tukey’s multiple-comparison test when an overall group effect was detected. Variables not satisfying parametric assumptions were analyzed using the Kruskal–Wallis test. Following a significant Kruskal–Wallis test, three prespecified pairwise comparisons addressing the primary biological hypotheses of the study (Control vs. CPF, CPF vs. CPF + CUR, and Control vs. CPF + CUR) were performed using two-sided exact Mann–Whitney U tests. These three comparisons constituted a single family (family size = 3), and Bonferroni-adjusted *p*-values were calculated accordingly. Appropriate effect-size estimates and their 95% confidence intervals (CIs) were reported together with exact *p*-values. Statistical significance was defined as *p* < 0.05. Statistical analyses were performed using SPSS version 21.0 (IBM Corp., Armonk, NY, USA).

## 3. Results

### 3.1. Litter Outcomes, Anogenital Distance, and Birth Weight

Litter size did not differ significantly among the five groups (Table 1; one-way ANOVA, *F* = 1.18, *p* = 0.351; Control 9.2 ± 1.3, CPF 7.8 ± 1.3, CPF + CUR 9.0 ± 0.7, CUR 8.6 ± 1.1, SHAM 9.4 ± 1.8 pups/litter; full statistics in Supplementary Table S1), nor did the proportion of female pups at birth (Table 1; Control 52.2%, CPF 43.6%, CPF + CUR 55.6%, CUR 48.8%, SHAM 42.6%). Neonatal mortality was confined to the first postnatal day: two pups died in the CPF group and one in the CPF + CUR group, with no further deaths in any group through PND28. Survival to PND28 was accordingly 94.9% (37/39) in CPF and 97.8% (44/45) in CPF + CUR, versus 100% in Control, CUR, and SHAM (Table 1); because the resulting contingency table had very small expected cell counts, the Fisher–Freeman–Halton exact test was used rather than chi-square, and the difference across groups was not statistically significant (*p* = 0.076).

Female pup birth weight was significantly lower in CPF than in Control (Table 1; Kruskal–Wallis, *H* = 19.26, *p* = 0.0007; CPF 5.00 [4.90–5.10] g vs. Control 6.20 [5.90–6.40] g, *p*_adj = 0.024) and was significantly higher with curcumin co-treatment than with CPF alone (CPF + CUR 5.50 [5.50–5.50] g, *p*_adj = 0.024 vs. CPF), although CPF + CUR did not differ significantly from Control after correction (*p*_adj = 0.095), consistent with partial recovery. Female AGD at PND1 followed a similar pattern (Table 1): CPF (1.390 [1.380–1.390] mm) was significantly shorter than Control (1.520 [1.500–1.580] mm; *p*_adj = 0.024), and CPF + CUR (1.400 [1.390–1.410] mm) remained significantly shorter than Control (*p*_adj = 0.024) with no significant difference from CPF (*p*_adj = 0.667), indicating that curcumin did not measurably normalize AGD itself. When AGD was expressed relative to body size as AGDi (Table 1), the omnibus difference across groups remained significant (one-way ANOVA, *F* = 3.55, *p* = 0.024), but none of the three prespecified pairwise comparisons survived Bonferroni correction (Control vs. CPF *p*_adj = 0.178; CPF vs. CPF + CUR *p*_adj = 0.983; Control vs. CPF + CUR *p*_adj = 0.066; full statistics in Supplementary Table S1)—the omnibus effect reflects the overall spread across all five groups rather than one dominant pairwise contrast, and no directional claim is made for AGDi at the pairwise level.

### 3.2. Body and Ovarian Weights and Gonadosomatic Index

Body weight at PND28 did not differ among groups (Table 2; one-way ANOVA, *F* = 0.16, *p* = 0.958), whereas absolute ovarian weight and the gonadosomatic index (GSI) were both significantly reduced by CPF exposure. Right ovarian weight was lower in CPF (13.00 [12.90–13.20] mg) than in Control (15.80 [15.70–16.00] mg; Kruskal–Wallis, *H* = 18.96, *p* = 0.0008; *p_adj* = 0.024), partially recovered with curcumin (CPF + CUR 14.60 [14.50–14.70] mg vs. CPF, *p*_adj = 0.024), but remained below Control (*p*_adj = 0.024). Left ovarian weight showed the same pattern with a larger effect size (one-way ANOVA, *F* = 76.26, *p* < 0.0001; all three pairwise comparisons significant, *p*_adj ≤ 0.0001), as did total ovarian weight (Kruskal–Wallis, *H* = 18.58, *p* = 0.0010; all three pairwise comparisons *p*_adj = 0.024; Table 2, full statistics in Supplementary Table S2). GSI was significantly lower in CPF than Control (0.0350 ± 0.0034% vs. 0.0416 ± 0.0035%; one-way ANOVA, *F* = 5.12, *p* = 0.0053; *p*_adj = 0.020), while the CPF + CUR value (0.0384 ± 0.0022%) did not differ significantly from either CPF or Control after correction (*p*_adj = 0.433 and 0.469, respectively). Because body weight was statistically indistinguishable across all five groups while both absolute ovarian weight and GSI were significantly and consistently reduced in CPF (Table 2), the ovarian phenotype is unlikely to be a secondary consequence of generalized growth impairment and instead indicates an ovary-directed effect of CPF exposure.

### 3.3. Ovarian Histopathological Findings

**Figure 1:**
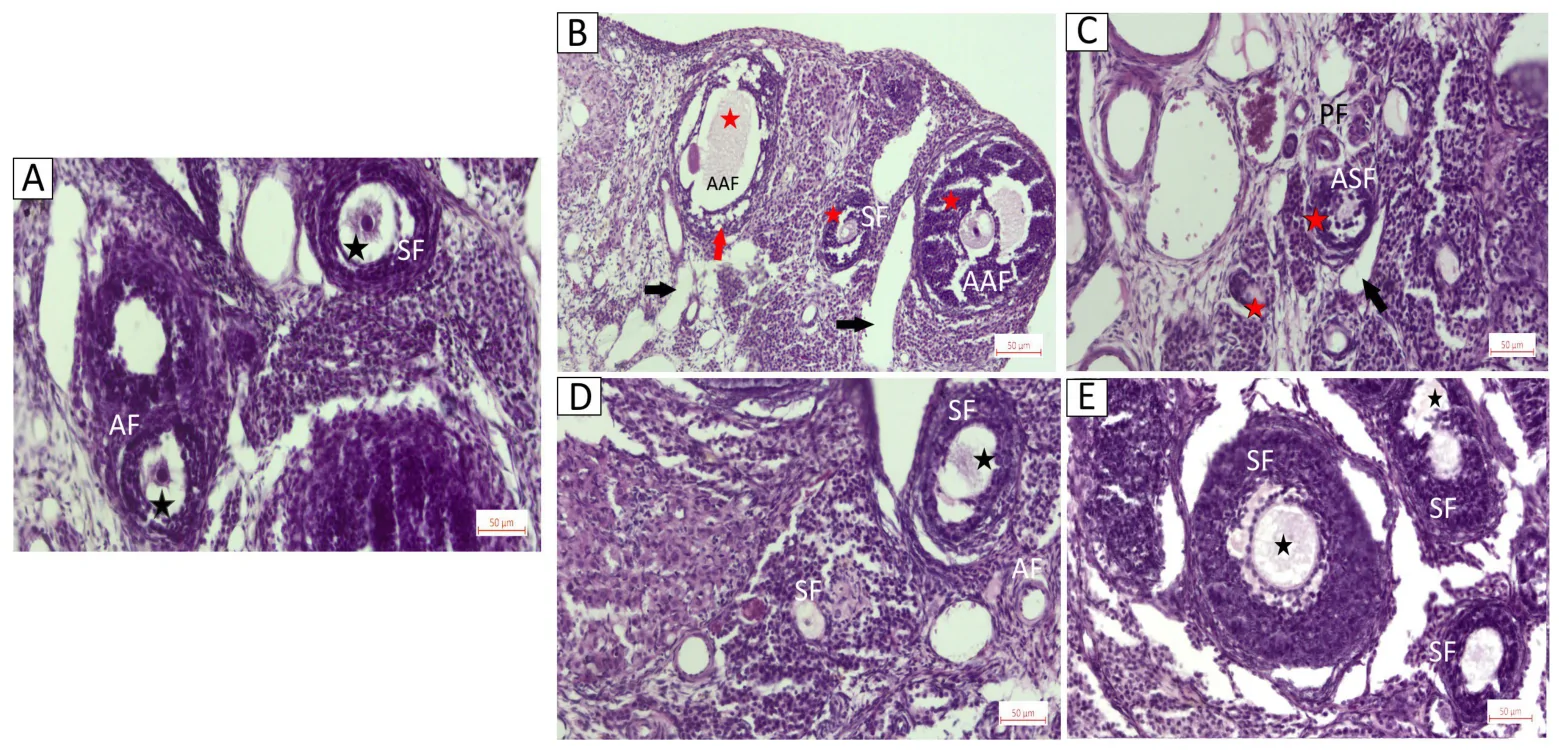
Representative H&E-stained ovarian sections from female rat offspring on postnatal day 28 (PND28): Control (**A**), CPF (**B**), CPF + CUR (**C**), CUR (**D**), and Sham (**E**). Black stars indicate healthy follicles; red stars indicate degenerated follicles; red arrows indicate granulosa cell layer detachment/disorganization; and black arrows indicate stromal disorganization. PF: primary follicle; SF: secondary follicle; AF: antral follicle; ASF: atretic secondary follicle; AAF: atretic antral follicle. Scale bars = 50 µm.

Representative ovarian micrographs are shown in Figure 1. Control ovaries retained the expected prepubertal organization: follicles and cortical boundaries were readily identifiable, the germinal epithelium formed a continuous cuboidal-to-prismatic layer, and the underlying fibrous tunica albuginea was distinct. Early-stage follicles were distributed within an orderly cortical stroma, with intact connective tissue between neighboring follicles. The medulla contained loose connective tissue, vessels of different calibres, and morphologically normal hilus-cell clusters.

The Sham and CUR groups closely resembled Control tissue, showing intact follicular profiles and no apparent disruption of stromal arrangement. CPF-exposed ovaries exhibited atretic follicles, detachment and disorganization of granulosa cell layers, stromal disorganization, and focal vascular congestion or haemorrhage. These alterations were less pronounced in the CPF + CUR group, in which preserved follicles coexisted with occasional degenerative follicular profiles and residual stromal disorganization.

**Figure 2:**
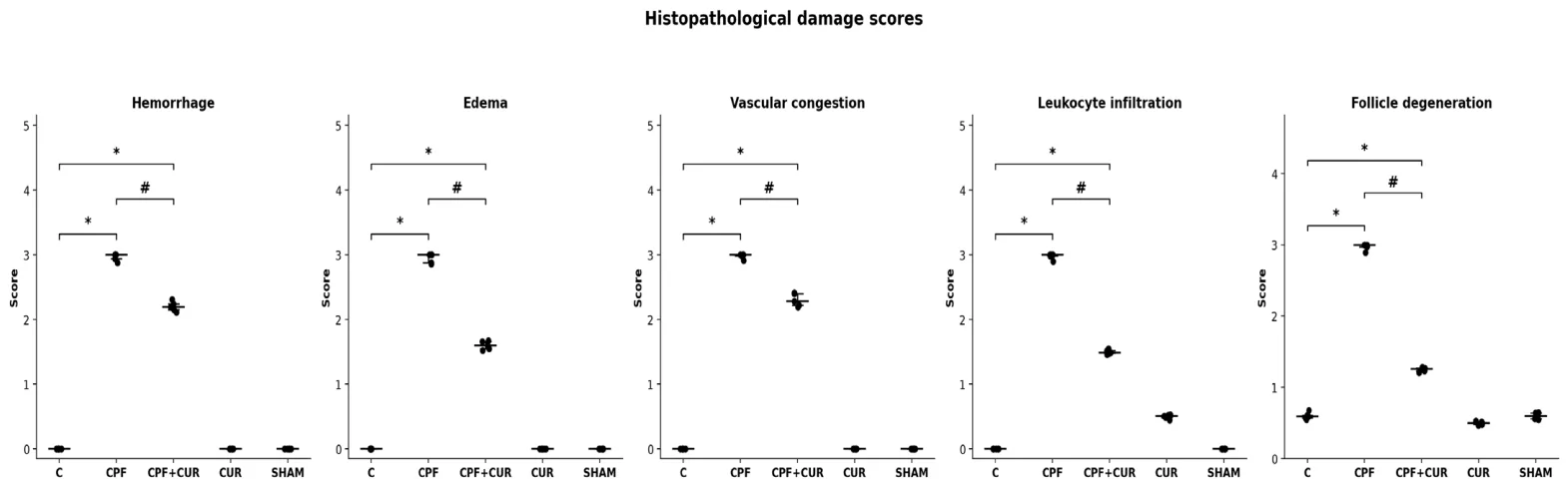
Histopathological damage scores in ovarian tissue after prenatal CPF exposure and/or curcumin administration. Values are presented as median (IQR); individual points represent litter-level observations (*n* = 5/group). * *p*_adj < 0.05 vs. Control; # *p*_adj < 0.05 vs. CPF. Detailed statistics are provided in Supplementary Table S7.

Ordinal histopathological damage scores (0–3 scale; reported as median [IQR]) were analyzed by Kruskal–Wallis with exact Mann–Whitney U pairwise tests regardless of distribution. All five scores—hemorrhage, edema, vascular congestion, leukocyte infiltration, and follicle degeneration—were significantly higher in CPF than Control (Figure 2; all omnibus *p* ≤ 0.0002) and were significantly reduced by curcumin co-treatment (all CPF-vs-CPF + CUR comparisons *p*_adj = 0.024), while remaining significantly above Control in CPF + CUR for every score (all *p*_adj = 0.024; full statistics in Supplementary Table S7). For hemorrhage, edema, vascular congestion, and leukocyte infiltration, CPF scores clustered at or near the maximum of the scale (median = 3.00), producing a ceiling effect that likely compresses the apparent magnitude of the CPF + CUR improvement for these four parameters (Figure 1; see Limitations). Follicle degeneration, which did not show this ceiling (Control baseline 0.59 [0.57–0.61] rather than 0.00), showed the clearest graded curcumin-associated improvement across the full 0–3 range.

### 3.4. Stereological Findings

#### 3.4.1. Follicle Numbers

**Figure 3:**
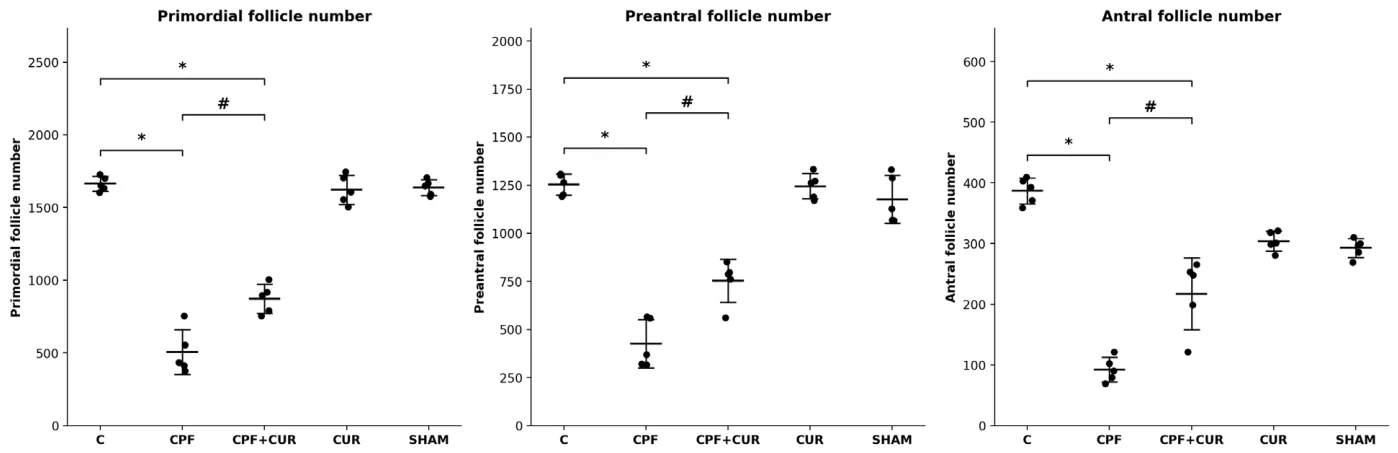
Ovarian follicle numbers (primordial, preantral, and antral) after prenatal CPF exposure and/or curcumin administration. Values are presented as mean ± SD; individual points represent litter-level observations (*n* = 5/group). * *p*_adj < 0.05 vs. Control; # *p*_adj < 0.05 vs. CPF. Detailed statistics are provided in Supplementary Table S3.

Primordial, preantral, and antral follicle counts were assessed in all five groups, including SHAM (Control 1664.0 ± 50.3, CPF 507.4 ± 154.4, CPF + CUR 872.9 ± 100.8, CUR 1623.0 ± 100.3, SHAM 1638.2 ± 53.9 for primordial follicles). Counts were all significantly reduced in CPF relative to Control (Figure 3; all omnibus one-way ANOVA *p* < 0.0001—primordial *F* = 145.81, preantral *F* = 65.89, antral *F* = 62.36), significantly increased by curcumin co-treatment relative to CPF (primordial *p*_adj = 0.0001; preantral *p*_adj = 0.0005; antral *p*_adj < 0.0001), and remained significantly below Control in CPF + CUR for every follicle class (all *p*_adj < 0.0001; full statistics in Supplementary Table S3).

#### 3.4.2. Ovarian Cortex and Medulla Volumes

**Figure 4:**
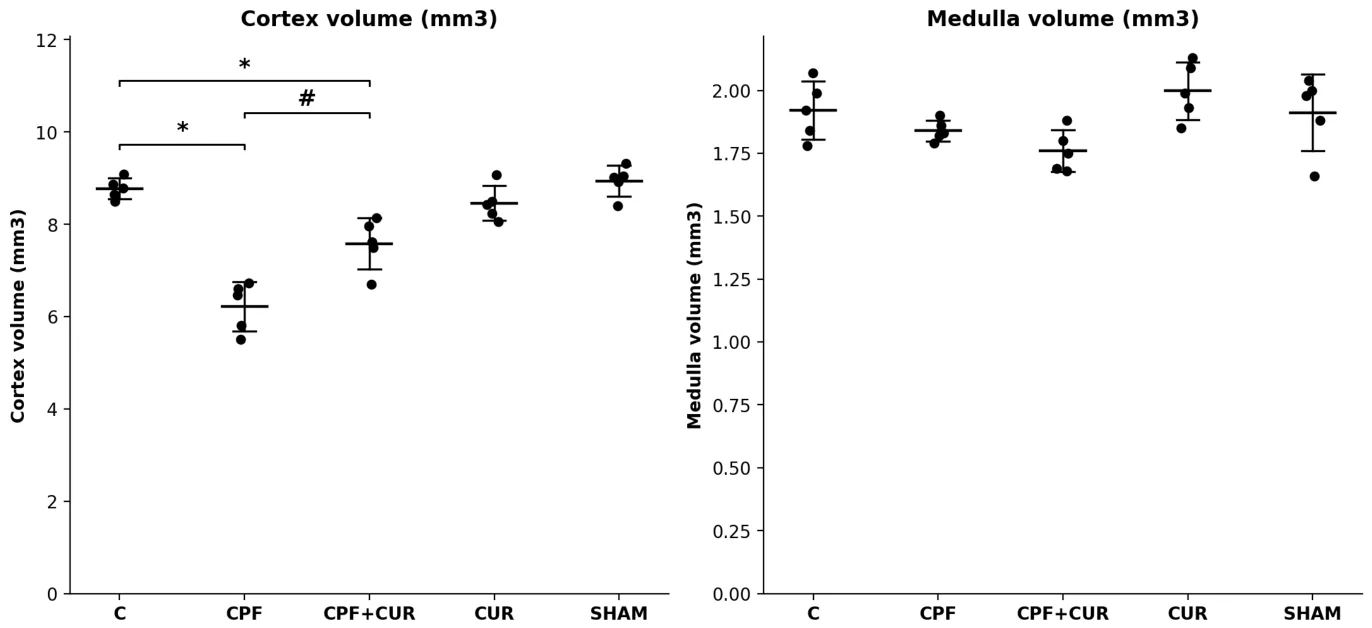
Ovarian cortex and medulla volumes after prenatal CPF exposure and/or curcumin administration. Values are presented as mean ± SD; individual points represent litter-level observations (*n* = 5/group). * *p*_adj < 0.05 vs. Control; # *p*_adj < 0.05 vs. CPF. Detailed statistics are provided in Supplementary Table S3.

Ovarian cortex volume was assessed in all five groups (Control 8.77 ± 0.23, CPF 6.22 ± 0.53, CPF + CUR 7.58 ± 0.56, CUR 8.46 ± 0.38, SHAM 8.94 ± 0.34 mm3) and was significantly reduced in CPF relative to Control, following the same graded curcumin-associated pattern as the follicle counts above (Figure 4; one-way ANOVA, *F* = 34.69, *p* < 0.0001; all three pairwise comparisons significant, *p*_adj ≤ 0.0022). Medulla volume also differed significantly across the five groups in the omnibus test (*F* = 3.46, *p* = 0.026), but no individual pairwise comparison reached Bonferroni-corrected significance (all *p*_adj ≥ 0.17; full statistics in Supplementary Table S3), so no directional claim is made for this parameter.

#### 3.4.3. Precision of the Stereological Estimates

For volume estimates, every CE was <0.05. Most follicle-count CEs were ≤0.05, and the remaining values did not exceed 0.06, remaining well within the commonly accepted <0.10 threshold. These values indicate adequate sampling precision; CE and CV results for individual outcomes are listed in Table 3.

### 3.5. Biochemical Findings

#### 3.5.1. Ovarian Oxidative Status

**Figure 5:**
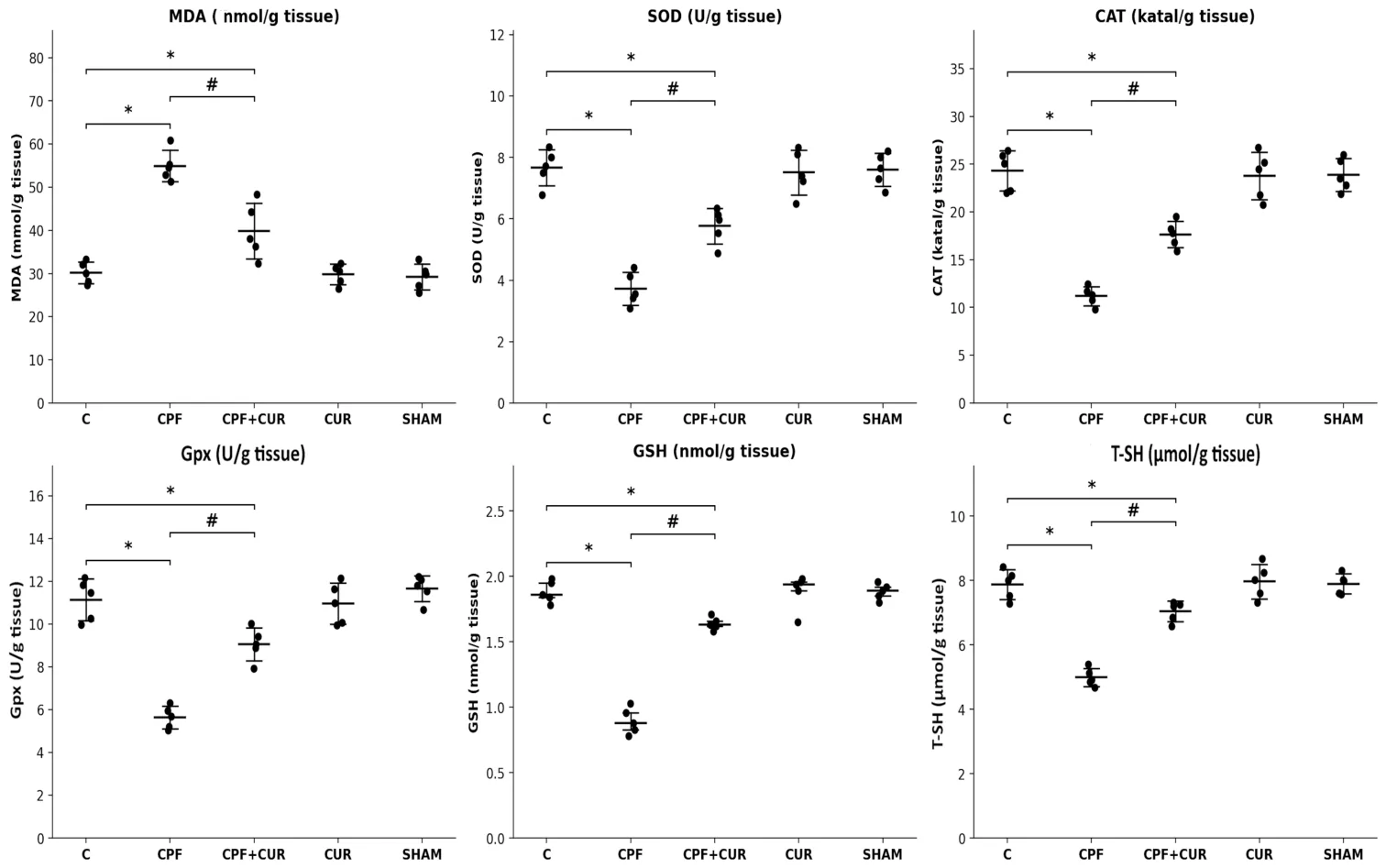
Ovarian oxidative stress and antioxidant status (MDA, SOD, CAT, GPx, GSH, T-SH) after prenatal CPF exposure and/or curcumin administration. Values are presented as mean ± SD (MDA, SOD, CAT, GPx, T-SH) or median (IQR) (GSH); individual points represent litter-level observations (*n* = 5/group). * *p*_adj < 0.05 vs. Control; # *p*_adj < 0.05 vs. CPF. Detailed statistics are provided in Supplementary Table S6.

CPF exposure significantly increased the lipid-peroxidation marker MDA and significantly decreased every measured antioxidant parameter (GSH, GPx, total thiols [T-SH], SOD, and CAT) relative to Control (Figure 5; all omnibus tests *p* < 0.0001). Curcumin co-treatment significantly reversed all six changes relative to CPF alone (all CPF-vs-CPF + CUR comparisons *p*_adj ≤ 0.0002), although CPF + CUR values remained significantly different from Control for every parameter (all *p*_adj ≤ 0.024; full statistics in Supplementary Table S6), indicating substantial but incomplete restoration of the oxidative balance.

#### 3.5.2. Serum Reproductive Hormones and Anti-Müllerian Hormone

Serum concentrations of estradiol, progesterone, prolactin, FSH and AMH were obtained as exploratory endpoints. Because these immunoassays were not analytically validated in-house (no in-house LOQ, no cross-reactivity profile, and no inter-assay CV; all samples were assayed on a single plate), the values are reported for descriptive purposes only and no quantitative treatment effect is inferred. Full group-level statistics are provided in Supplementary Table S4, and the available kit specifications and intra-assay precision, together with the outstanding validation items, are compiled in Supplementary Table S9.

Descriptively, the direction of change paralleled the ovarian findings: relative to Control, the CPF group showed lower estradiol, progesterone and AMH and higher FSH, whereas prolactin was similar across groups; in the CPF + CUR group these differences were attenuated. For AMH, group means were 1.08 ± 0.13 (Control), 0.52 ± 0.08 (CPF) and 0.82 ± 0.08 ng/mL (CPF + CUR), a pattern consistent with the reduced follicle numbers reported in Section 3.4.1, although the CPF + CUR value remained below Control. These observations require confirmation with analytically validated, matrix-appropriate assays.

Two measurement caveats apply. First, absolute serum progesterone values (13–27 ng/mL) exceed the physiological range expected for prepubertal female rats and most likely reflect assay cross-reactivity rather than true circulating progesterone; the identity of the measured analyte is therefore uncertain. Second, AMH immunoassays are not harmonised across platforms and absolute values are assay-dependent; AMH was measured with the CUSABIO CSB-E11162r rat-specific ELISA (working range 0.2–15 ng/mL, sensitivity 0.1 ng/mL), so the Control value of 1.08 ng/mL lies within the mid-range of the standard curve and is not directly comparable with reference ranges obtained on other assays. Both points are detailed in the Limitations and in Supplementary Tables S4 and S9.

#### 3.5.3. Ovarian NF-κB and Pro-Inflammatory Cytokines

**Figure 6:**
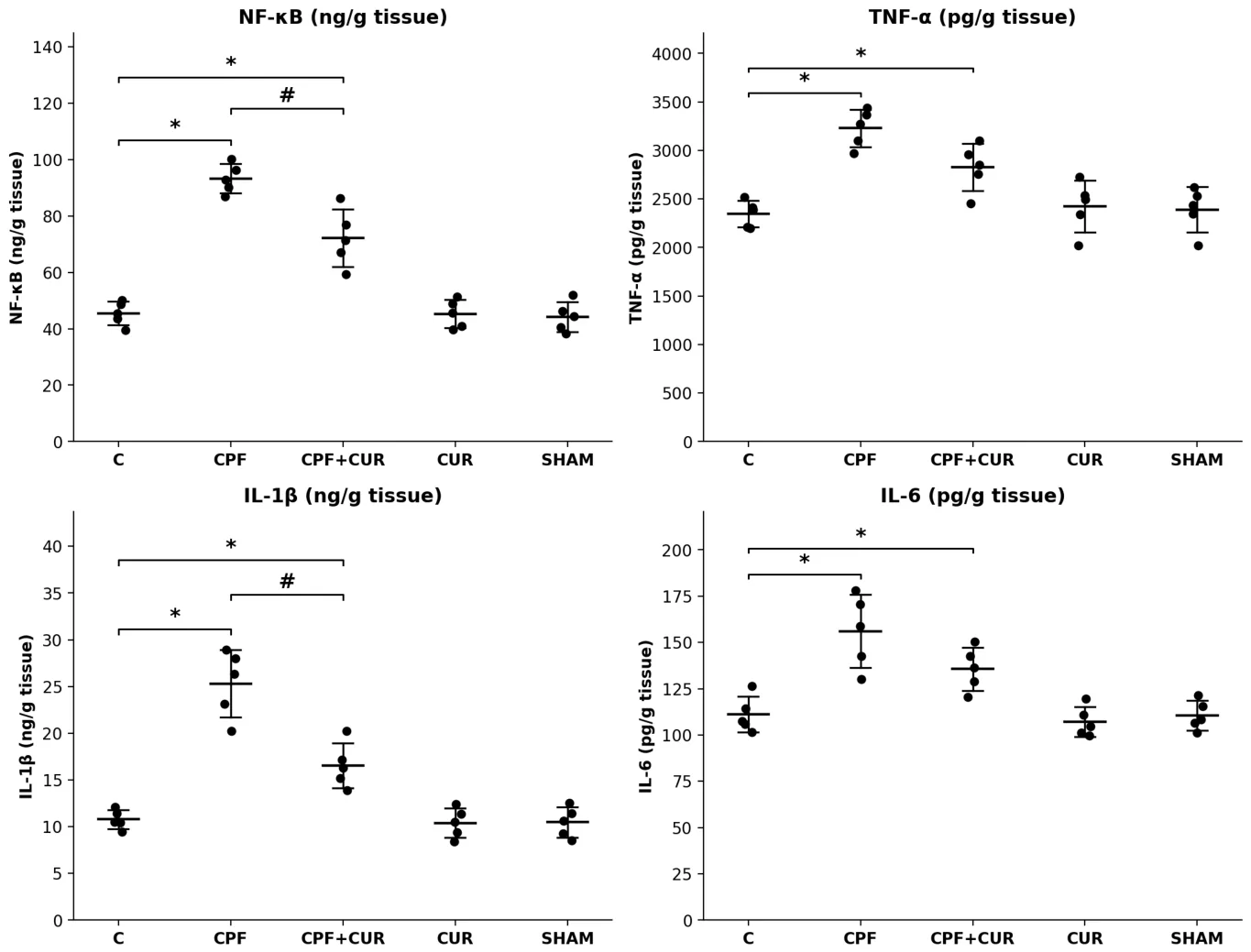
Ovarian NF-κB and pro-inflammatory cytokine levels (TNF-α, IL-1β, IL-6) after prenatal CPF exposure and/or curcumin administration. Values are presented as mean ± SD; individual points represent litter-level observations (*n* = 5/group). * *p*_adj < 0.05 vs. Control; # *p*_adj < 0.05 vs. CPF. Detailed statistics are provided in Supplementary Table S5.

All four inflammatory mediators were significantly elevated by CPF exposure relative to Control (NF-κB, IL-1β, IL-6, TNF-α; Figure 6; all omnibus one-way ANOVA *p* < 0.0001). Curcumin co-treatment significantly attenuated NF-κB (*p*_adj = 0.0003 vs. CPF) and IL-1β (*p*_adj < 0.0001 vs. CPF), both of which nevertheless remained significantly above Control in CPF + CUR (*p*_adj < 0.0001 for both). TNF-α and IL-6 showed the same directional reduction with curcumin, but the CPF-vs-CPF + CUR comparison did not reach Bonferroni-corrected significance for either marker (TNF-α *p*_adj = 0.058; IL-6 *p*_adj = 0.104), while both remained significantly elevated versus Control in CPF + CUR (TNF-α *p*_adj = 0.019; IL-6 *p*_adj = 0.034; full statistics in Supplementary Table S5).

#### 3.5.4. Serum Cholinesterase Activity

**Figure 7:**
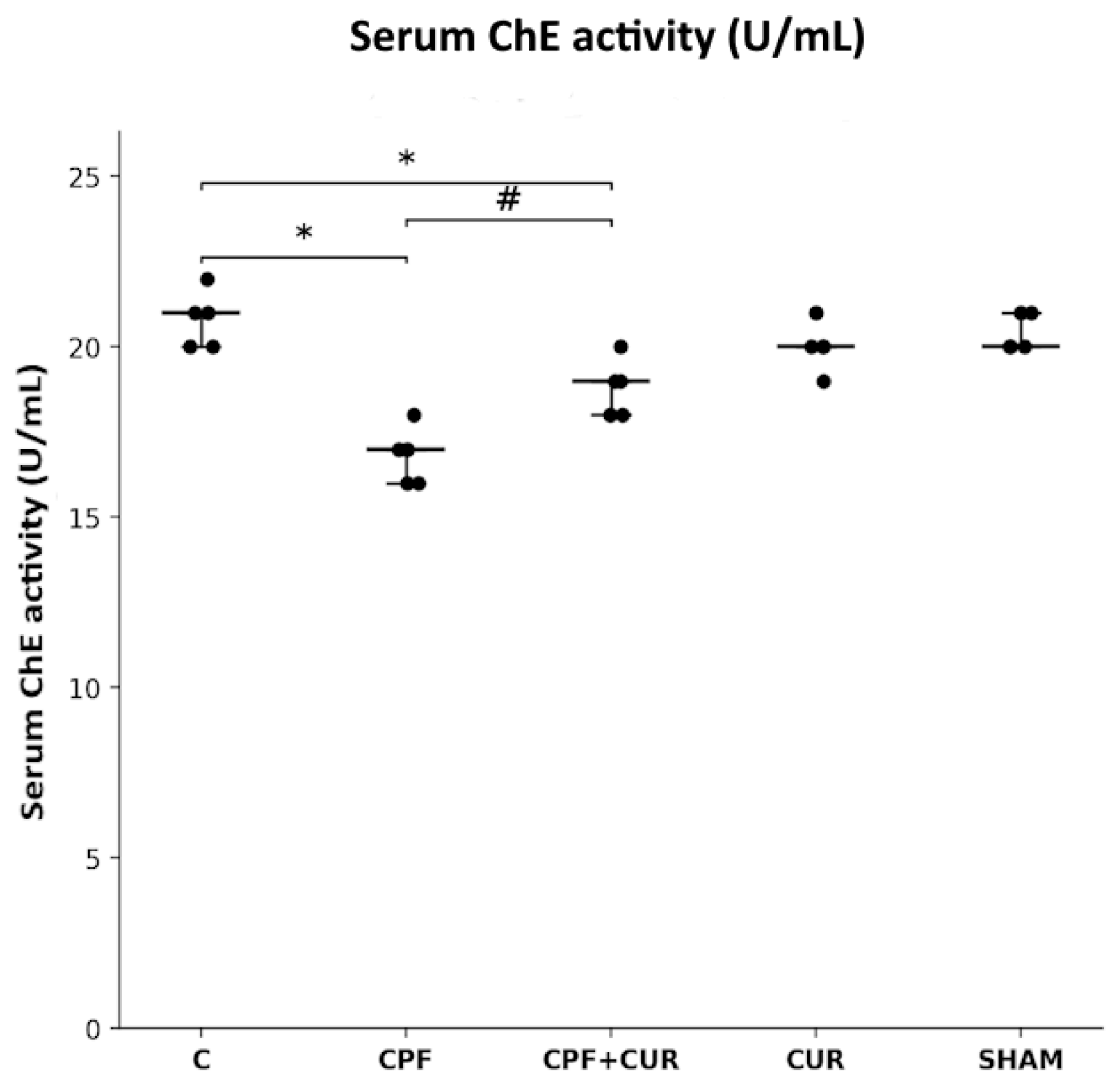
Serum cholinesterase (ChE) activity after prenatal CPF exposure and/or curcumin administration. Values are presented as median (IQR); individual points represent litter-level observations (*n* = 5/group). * *p*_adj < 0.05 vs. Control; # *p*_adj < 0.05 vs. CPF. Detailed statistics are provided in Supplementary Table S8.

Serum ChE activity was significantly lower in CPF than Control (17.0 [16.0–17.0] U/mL vs. 21.0 [20.0–21.0] U/mL; Figure 7; Kruskal–Wallis, *H* = 18.24, *p* = 0.0011; *p*_adj = 0.024), consistent with the known mechanism of organophosphate action. Curcumin co-treatment significantly increased ChE activity relative to CPF alone (CPF + CUR 19.0 [18.0–19.0] U/mL, *p*_adj = 0.048), although activity remained significantly below Control (*p*_adj = 0.048; full statistics in Supplementary Table S8), indicating partial but incomplete restoration.

## 4. Discussion

One of the major findings of the present study was the significant reduction in gonadosomatic index (GSI) observed in the CPF group. GSI is considered an important indicator of gonadal development and reproductive status. In rodent and human studies, prenatal or developmental exposure to pesticides, including CPF, has been reported to reduce body and ovarian weights and impair reproductive development [11,32,33]. The decrease in GSI observed in the present study may therefore be associated with CPF-induced developmental and structural alterations in ovarian tissue. In the curcumin co-treatment group, GSI values were relatively higher; given the partial nature of this change, it should be interpreted cautiously, although broadly in line with previous reports on curcumin in ovarian and reproductive tissues [9,34]. At the maternal level, CPF reduced gestational weight gain, and hepatic histopathological alterations in the same experimental cohort have been reported previously [35], indicating concurrent maternal toxicity at this dose. Notably, the CPF dose used (5 mg/kg/day) lies within the range applied in previous developmental studies but above current regulatory NOAEL/BMD-based limits; accordingly, the present findings characterize developmental ovarian toxicity under an experimental exposure and are not directly generalizable to typical human environmental exposure. Importantly, body weight at PND28 did not differ among the experimental groups, whereas right, left, and total ovarian weights were significantly reduced following CPF exposure. Thus, the reduction in ovarian mass and GSI cannot be attributed to lower overall body weight at PND28 and supports an ovary-directed effect of prenatal CPF exposure.

Histopathological evaluation revealed that CPF exposure induced marked structural alterations in ovarian tissue, including follicular degeneration, leukocyte infiltration, and stromal disorganization. Similar histopathological findings have previously been reported following CPF exposure in ovarian tissues [3,9,11,12]. Histopathological damage was significantly attenuated by curcumin co-administration compared with CPF alone; however, lesion scores remained higher than those in the Control group, indicating a partial modulatory rather than a complete protective effect, broadly consistent with previous reports on curcumin in different experimental models [9,18]. For four of the five scored parameters, CPF-group values clustered near the upper limit of the 0–3 scale, a ceiling effect that likely narrowed the apparent margin of curcumin’s protective effect for those parameters specifically (see Limitations).

Stereological analyses demonstrated significant reductions in primordial, preantral, and antral follicle numbers following prenatal CPF exposure. Both cortical and medullary ovarian volumes also differed significantly across groups in the omnibus test. The reduction in follicular populations may reflect increased follicular degeneration and disruption of follicular organization following prenatal CPF exposure. Previous studies have shown that pesticide exposure may impair folliculogenesis and increase follicular atresia through oxidative and inflammatory mechanisms [7,13,36]. Curcumin co-administration significantly increased primordial, preantral, and antral follicle numbers and cortical volume compared with CPF alone; however, these parameters remained significantly different from Control values, indicating only partial structural recovery. In contrast, no individual pairwise comparison reached statistical significance for medullary volume, and this compartment showed no numerical evidence of a curcumin-associated increase. This compartment-specific pattern suggests that the effects of curcumin on ovarian architecture may not be uniform across cortical and medullary regions and should therefore be interpreted cautiously.

Biochemical analyses demonstrated increased oxidative stress and inflammatory markers in the CPF group. Elevated MDA, NF-κB, TNF-α, IL-1β, and IL-6 levels together with reduced antioxidant enzyme activities suggest that CPF exposure may alter oxidative and inflammatory balance in ovarian tissue. Previous experimental studies similarly reported increased oxidative stress and inflammatory responses following CPF exposure [7,9,11]. Curcumin co-administration significantly attenuated the CPF-induced alterations in oxidative stress markers and in NF-κB and IL-1β; the reduction in TNF-α and IL-6 with curcumin followed the same direction but did not reach statistical significance for this comparison. Across all inflammatory and oxidative parameters, values in the CPF + CUR group remained significantly different from Control, indicating partial rather than complete normalization of ovarian redox and inflammatory status. This modulatory effect may be related to the antioxidant and anti-inflammatory properties of curcumin and is broadly consistent with previous experimental reports [9,15,18].

Serum cholinesterase activity was significantly lower in the CPF group than in controls at PND28. This observation should be interpreted with caution. Because gestational exposure ended at parturition, approximately four weeks before measurement, and because cholinesterase is restored by resynthesis over a period of days to weeks, a persistent reduction in blood cholinesterase at this interval is not readily explained by direct enzyme inhibition and remains an unexplained finding that requires confirmation. Prior work reporting long-term cholinergic changes after developmental organophosphate exposure refers primarily to central (brain) receptor and signalling endpoints, including lasting developmental effects on the cholinergic system [37], and renewed cholinergic deficits emerging later in development [38], rather than to persistent cholinesterase inhibition in blood, and therefore does not directly account for the present serum finding. A limited contribution of lactational transfer from residual maternal body burden, or of measurement variability, cannot be excluded. Confirmation in an appropriate matrix (erythrocyte or brain acetylcholinesterase) would be required to establish the nature of this change. Serum cholinesterase activity was higher in the CPF + CUR group than in the CPF group, although it remained below Control. As noted above, the persistence of this reduction at PND28 is itself unexplained; this group pattern is therefore described only as a parallel observation and is not interpreted mechanistically.

Serum AMH—produced by granulosa cells of growing follicles and a recognised marker of the ovarian follicle pool [39]—was measured as an exploratory endpoint (Supplementary Table S4). Its group-level pattern paralleled the stereological findings, with lower values in the CPF group and intermediate values with curcumin co-treatment. Because this assay was not analytically validated, AMH is interpreted only as a descriptive observation supporting the follicle-count results, not as a quantitative endpoint.

Prenatal CPF exposure was associated with a reduction in absolute anogenital distance (AGD) at PND1. This finding is broadly consistent with previous evidence suggesting that developmental exposure to organophosphorus insecticides may alter AGD. In rats, developmental exposure to chlorpyrifos-methyl was associated with reduced absolute but not relative AGD in F1 offspring, indicating that differences in body size may contribute to apparent changes in AGD [40]. More recently, a human birth cohort examining prenatal chlorpyrifos exposure similarly reported a modest, although non-significant, tendency toward shorter AGD in both female and male offspring [41]. In the present study, neonatal body weight was also reduced, and normalization of AGD to body size using the anogenital distance index (AGDi) eliminated significant differences in the primary pairwise comparisons. Therefore, the reduction in absolute AGD should be interpreted cautiously and may, at least in part, reflect differences in neonatal growth rather than an endocrine-disrupting effect independent of body size.

The present study also observed group-level differences in serum reproductive hormone concentrations following prenatal CPF exposure. As exploratory endpoints (Section 3.5.2, Supplementary Table S4), these are described only descriptively: relative to Control, the CPF group showed lower estradiol and progesterone and higher FSH, whereas prolactin was numerically lower without a clear separation between groups. Previous studies have similarly associated CPF and other pesticide exposures with disruption of reproductive hormone profiles; however, the direction and magnitude of individual hormonal changes appear to depend on the pesticide, dose, exposure window, developmental stage, and reproductive status [13,42]. Because all evaluations were performed at postnatal day 28, corresponding to a prepubertal developmental stage, and because these assays were not analytically validated, the hormonal findings should be interpreted primarily as relative between-group patterns within the same assay rather than as definitive physiological hormone concentrations or indicators of long-term reproductive function.

### Limitations

This study has several limitations. All evaluations were performed at a single prepubertal time point (PND28); the findings therefore reflect early ovarian alterations rather than long-term reproductive outcomes, and the durability of the curcumin-associated effects into adulthood was not assessed.

The hormone measurements have important analytical limitations. Serum reproductive hormones and AMH were quantified by commercial ELISA and were not confirmed with a reference method such as LC-MS/MS. These assays were not validated in-house: an in-house limit of quantification, a cross-reactivity profile, and an inter-assay coefficient of variation are not available, the latter because all samples were assayed on a single plate. A request for additional validation data was not answered by the manufacturer, which does not substitute for in-house validation. In particular, absolute serum progesterone values exceeded the range expected for prepubertal females and most likely reflect assay cross-reactivity rather than true circulating concentrations. For these reasons, all serum hormone and AMH data are reported as exploratory (Supplementary Tables S4 and S9) and were interpreted only as relative between-group patterns, not as validated quantitative endpoints.

Cholinesterase activity was assessed in serum with an assay that does not discriminate acetylcholinesterase from butyrylcholinesterase, and the reduction observed at PND28—four weeks after exposure ended—could not be confirmed in a conventional matrix (erythrocyte or brain acetylcholinesterase); this finding should therefore be regarded as preliminary and requires confirmation.

Additional limitations apply to the biochemical and design aspects of the study. Ovarian NF-κB was quantified as total tissue protein rather than as its activated (phosphorylated) form, and apoptosis markers were not assessed, so the inflammatory findings should be regarded as indicative rather than mechanistic. Finally, although the litter was used as the experimental unit to avoid litter effects, the number of litters per group was limited; confirmation in larger cohorts and at later developmental stages is warranted.

## 5. Conclusions

When the stereological, histopathological, and biochemical findings were evaluated together, prenatal CPF exposure was associated with marked ovarian alterations at PND28, including reduced follicular populations, structural tissue injury, oxidative stress, and inflammatory activation. The reductions in primordial, preantral, and antral follicle numbers, together with alterations in ovarian morphology and AMH levels, support an adverse effect of prenatal CPF exposure on the developing ovarian follicular compartment.

Curcumin co-administration significantly attenuated many of the CPF-induced histopathological, stereological, oxidative, and inflammatory alterations; however, recovery was incomplete and was not consistent across all outcomes. Thus, the present findings support a partial modulatory rather than a complete protective effect of curcumin on CPF-induced developmental ovarian toxicity. Although curcumin has been reported to have potential benefits for human female reproductive health [17], these findings are limited to an experimental rat model and should not be directly extrapolated to humans.

## Figures and Tables

**Table 1:** Litter outcomes, female birth weight, anogenital distance (AGD), and anogenital distance index (AGDi).

Group	Litters (*n*)	Litter Size (Mean ± SD)	Male/Female at Birth	Female (%)	Survival to PND28 (%)	Female Birth Weight at PND1 (g)	Female AGD at PND1 (mm)	AGDi (mm/g^1/3^)
Control	5	9.20 ± 1.30	22/24	52.2	100.0 (46/46)	6.20 (5.90–6.40)	1.520 (1.500–1.580)	0.840 ± 0.032
CPF	5	7.80 ± 1.30	22/17	43.6	94.9 (37/39)	5.00 (4.90–5.10) *	1.390 (1.380–1.390) *	0.803 ± 0.026
CPF + CUR	5	9.00 ± 0.71	20/25	55.6	97.8 (44/45)	5.50 (5.50–5.50) #	1.400 (1.390–1.410) *	0.794 ± 0.019
CUR	5	8.60 ± 1.14	22/21	48.8	100.0 (43/43)	6.30 (6.30–6.40)	1.550 (1.510–1.580)	0.838 ± 0.024
Sham	5	9.40 ± 1.82	27/20	42.6	100.0 (47/47)	6.00 (6.00–6.10)	1.510 (1.480–1.550)	0.832 ± 0.026
Overall *p*		ANOVA, *p* = 0.3511			Fisher–Freeman–Halton exact, *p* = 0.0756	Kruskal–Wallis, *p* = 0.0007	Kruskal–Wallis, *p* = 0.0010	ANOVA, *p* = 0.0240

Values are mean ± SD or median (IQR), selected per outcome based on Shapiro–Wilk/Levene testing; corresponding statistical tests and the Bonferroni correction (family = 3) are detailed in Section 2.8. * *p*_adj < 0.05 vs. Control; # *p*_adj < 0.05, CPF vs. CPF + CUR. Male/Female at birth and Female (%) are descriptive counts only. See Section 3.1 for the calculation and statistical test used for Survival to PND28.

**Table 2:** Body weight, absolute ovarian weights, total ovarian weight, and gonadosomatic index (GSI) of female offspring at PND28.

Group	Body Weight at PND28 (g)	Right Ovary (mg)	Left Ovary (mg)	Total Ovarian Weight (mg)	GSI (%)
**Control**	76.98 ± 6.35	15.80 (15.70–16.00)	16.02 ± 0.30	31.90 (31.70–32.10)	0.0416 ± 0.0035
**CPF**	74.06 ± 7.36	13.00 (12.90–13.20) *	12.80 ± 0.19 *	25.90 (25.80–26.00) *	0.0350 ± 0.0034 *
**CPF + CUR**	76.40 ± 6.46	14.60 (14.50–14.70) * #	14.68 ± 0.56 *#	29.10 (29.00–30.00) * #	0.0384 ± 0.0022
**CUR**	76.06 ± 5.44	16.00 (15.90–16.10)	15.90 ± 0.21	31.90 (31.60–32.10)	0.0421 ± 0.0028
**Sham**	75.82 ± 5.31	16.00 (15.80–16.00)	15.82 ± 0.36	31.90 (31.50–31.90)	0.0420 ± 0.0032
**Overall *p***	ANOVA, *p* = 0.9580	Kruskal–Wallis, *p* = 0.0008	ANOVA, *p* ≤ 0.0001	Kruskal–Wallis, *p* = 0.0010	ANOVA, *p* = 0.0053

Values are mean ± SD or median (IQR), selected per outcome based on Shapiro–Wilk/Levene testing; corresponding statistical tests and the Bonferroni correction (family = 3) are detailed in Section 3.2. * *p*_adj < 0.05 vs. Control; # *p*_adj < 0.05, CPF vs. CPF + CUR. GSI (%) = [total ovarian weight (mg)/body weight (mg)] × 100 (Section 2.4).

**Table 3:** Sampling precision (CE) and between-animal variability (CV) for follicle-number and ovarian-volume estimates. C, Control; CPF, chlorpyrifos; CUR, curcumin.

	C CE	C CV	CPF CE	CPF CV	CPF + CUR CE	CPF + CUR CV	CUR CE	CUR CV	Sham CE	Sham CV
Primordial follicle number	0.04	0.23	0.06	0.26	0.05	0.31	0.04	0.27	0.03	0.3
Preantral follicle number	0.03	0.27	0.04	0.36	0.06	0.34	0.04	0.21	0.05	0.38
Antral follicle number	0.06	0.21	0.05	0.32	0.06	0.27	0.06	0.19	0.04	0.28
Ovarian cortex volume	0.02	0.15	0.02	0.21	0.02	0.19	0.01	0.13	0.01	0.15
Ovarian medulla volume	0.01	0.2	0.02	0.27	0.02	0.32	0.01	0.3	0.02	0.28

## Data Availability

The raw data supporting the conclusions of this article will be made available by the authors on request.

## Supplementary Materials

The following supporting information can be downloaded at: https://www.mdpi.com/article/10.3390/toxics14090820/s1, Table S1: Litter outcomes, birth weight, AGD, AGDi—full statistics for Table 1; Table S2: PND28 body weight, ovarian weights, GSI—full statistics for Table 2; Table S3: Ovarian follicle counts and stereological volumes—full statistics; Table S4: Reproductive hormones and AMH—full statistics (exploratory; not analytically validated in-house); Table S5: Ovarian inflammatory markers—full statistics; Table S6: Ovarian oxidative stress markers—full statistics; Table S7: Histopathological damage scores (ordinal)—full statistics; Table S8: Serum cholinesterase activity—full statistics; Table S9: Immunoassay validation data—kit specifications, intra-assay precision, and outstanding validation items.
